# Dyslipidemia impairs collateral artery formation after hindlimb ischemia: Adding insult to injury

**DOI:** 10.1016/j.jvssci.2024.100204

**Published:** 2024-04-05

**Authors:** Ali H. Hakim, Luke Brewster

**Affiliations:** a Division of Vascular Surgery, Department of Surgery, University of Nebraska Medical Center, Omaha, NE; b Division of Vascular Surgery, Department of Surgery, Emory University, Atlanta, GA; c Research and Surgical Services, Atlanta VA Medical Center, Decatur, GA

Peripheral arterial disease (PAD) is a complex disease encompassing numerous pathophysiologic mechanisms. Progressive atherosclerotic plaque formation and/or thrombosis from inflamed endothelium occur in a perturbed hemodynamic and atherogenic environment. To add insult to injury, the atherogenic environment impairs collateral formation.^1^^,^^2^

Under desirable conditions, blood flow at areas of blockage enlarges existing arterial networks (arteriogenesis) to provide important collateral blood flow. This has been demonstrated in patients and large animal models.^3^^,^^4^ Given that initial therapies—including structured exercise—aim to enhance collateralization, numerous studies have investigated the key mechanisms in both collateral artery growth and the formation of new vessels (angiogenesis) that can inosculate into existing collateral networks.^5^^,^^6^ Unfortunately, atherogenic and metabolic syndrome conditions impair arteriogenesis.^7^^,^^8^

In this issue, Yan et al^9^ investigate the effect of hyperlipidemia on collateral formation. Using multiple mouse models of hindlimb ischemia and hematopoietic stem cell (HSC) transplantation, they demonstrate that hyperlipidemia downregulates a ten-eleven translocation-1 (Tet1)-dependent mechanism, which restricts conversion of proinflammatory Ly6C^hi^ monocytes to proangiogenic Ly6C^low^ monocytes. This impairs collateral networks after hindlimb ischemia in these murine models. They demonstrate that this phenomenon is dependent of HSCs. They also demonstrate that knockout of Tet1 causes similar effects and that overexpression of Tet1 corrects these hyperlipidemic defects. The authors also investigate the effect of Tet1 on HSC differentiation gene expression in monocytes and HSCs and their regulation with DNA methylation. They show that DNA methylation and gene expression is altered by Tet1 at the HSC stage and persists through monocyte differentiation. Finally, they demonstrate improved perfusion recovery with the infusion of peripheral blood Ly6C^low^ monocytes 24 hours after hindlimb induction, thereby demonstrating the importance of the innate immune system in real-time arteriogenesis. This “hematologic bypass” is a promising approach to growing better collateral networks in atherogenic patients.

However, the translation of this work to patients requires further testing. The murine models used have some differences to a more chronic process seen in PAD patients, in whom the temporal changes of the innate immune system are not as well understood. There is also a possibility that some important pathways and cell chat occur alongside Ly6C^low^ monocytes. This can be discovered with a more comprehensive gene array or single cell analysis. Furthermore, the functional recovery of these animals is not yet known. There have been examples in the literature of improved perfusion but not function.^10^ Similarly, in PAD patients with abnormal ankle brachial indexes, higher abnormal indexes do not always correlate with better function.^11^ Thus, it will be exciting to see whether the improved perfusion outcomes demonstrated in their study can deliver better functional readouts. Our patients will be waiting.

*The opinions or views expressed in this commentary are those of the authors and do not necessarily reflect the opinions or recommendations of the JVS: Vascular Science or the Society for Vascular Surgery.*

## Disclosures

None.
